# Comparison of Alveolar Bone Thickness, Sagittal Root Positions, and Arch Forms in Class I, II, and III Malocclusions: A Cephalometric Study

**DOI:** 10.7759/cureus.37272

**Published:** 2023-04-07

**Authors:** Sumaiyya Haneen, Rajesh RNG, Anadha N Gujar, Rony Kondody

**Affiliations:** 1 Department of Orthodontics, Sri Rajiv Gandhi College of Dental Sciences, Bengaluru, IND; 2 Orthodontics and Dentofacial Orthopaedics, Sri Rajiv Gandhi College of Dental Sciences and Hospital, Bengaluru, IND

**Keywords:** tooth inclination, sagittal root position, morphology, cephalometry, arch shape, arch form, alveolar bone

## Abstract

Objective: This retrospective study aimed to evaluate the sagittal root position, arch shapes, and alveolar bone thickness in classes I, II, and III of skeletal patterns.

Material and methods: Alveolar bone thickness, sagittal root placements, and arch morphologies in classes I, II, and III malocclusions were measured using 30 study models and 30 lateral cephalograms, both with a mean age of 16.5 years. Based on their sagittal relations, 30 participants were classified into three groups each (classes I, II, and III). The ANOVA test was used to calculate the results.

Results: Between the three classes, there were statistically significant differences in the AP jaw relationship and root placements. Sagittal root location and dental arch shape showed no statistically significant correlation.

Conclusion: There was no correlation between the dental arch form and sagittal root position, and classes II and III were found to have higher mandibular incisor inclination. On all levels, oval arch forms were prevalent.

## Introduction

A lateral cephalogram is frequently used to assess the inclination of the central incisors as part of the orthodontic diagnosis and treatment planning process [1]. The central incisors' angles and how they relate to the alveolar bone around them are frequently the deciding elements in treatment choices for anterior-posterior incisor movements such as protrusion, proclination, retroclination, and retrusion. The cortical surfaces of alveolar bone represent a limiting factor for orthodontic tooth movement beyond which fenestration and/or dehiscence might occur. Defining anatomic boundaries for safe incisor root movements within each jaw can inform treatment decisions aimed at mitigating iatrogenic sequelae [2].

The alveolar bone's cortical surfaces serve as a physical barrier to orthodontic tooth movement. Any movement beyond this might have devastating repercussions, such as dehiscence or fenestration [3].

Ectopic tooth eruption is more likely to occur in crowded arches, which affects the thickness of the alveolar bone that encloses the roots of teeth [4]. Malocclusions, which may be linked to improper myofunctional behaviours, natural dental compensations, and other environmental effects, can negatively affect the root locations of incisors inside the alveolar processes [5].

The link between alveolar bone thickness, sagittal root placements, and arch shapes has received little attention in the literature on orthodontics [6]. Thus, utilising lateral cephalograms, the goals and objectives of our study were to compare the alveolar bone thickness, sagittal root placements, and arch morphologies in classes I, II, and III malocclusions.

## Materials and methods

The study was carried out at our institution's Department of Orthodontics and Dentofacial Orthopaedics. Lateral cephalograms of 30 people who visited the department seeking orthodontic treatment were taken. The sample size was calculated by using Daniel’s formula. Using wits evaluation, the participants were further categorised into classes I, II, and III groups based on the type of sagittal relationship.

All the cephalogram tracings were done manually on clear acetate sheets using a 2H pencil. The maxilla, mandible, maxillary, and mandibular first molars, as well as the maxillary and mandibular central incisors, were among the structures evaluated. Additionally recognised and traced were the occlusal planes, incisal edges, root apices, long axes of maxillary and mandibular incisors, the cementoenamel junction (CEJ) of maxillary incisors, and the midpoint of the maxillary incisor root (midway down the tooth's long axis between the CEJ and the apex).

**Figure 1:**
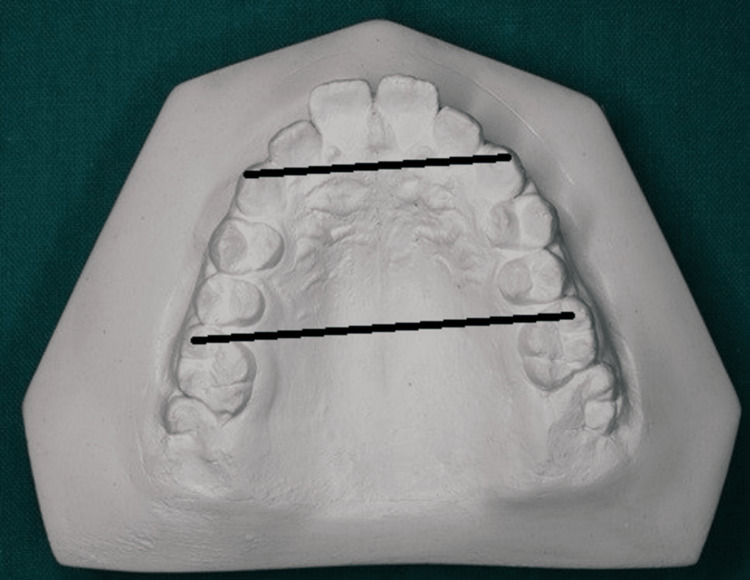
Inter-canine and inter-molar width

Measurements of the inter-canine width (ICW), canine depth (CD), inter-molar width (IMW), and molar depth were used to determine the arch form for all models (MD) (Figure 1).

These findings led to the calculation of the arch-form ratio.

According to Budiman's analysis, the arch shape ratio was obtained [7]. Each cast was then divided into three groups based on their ratio, as will be stated below: square, ovoid, and tapered. (a) An arch form ratio <45.30% denotes a square arch form. (b) An arch form ratio between 45.30% and 53.37% denotes an oval arch form. (c) An arch form ratio of more than 53.37% denotes a tapered arch form.

**Figure 2:**
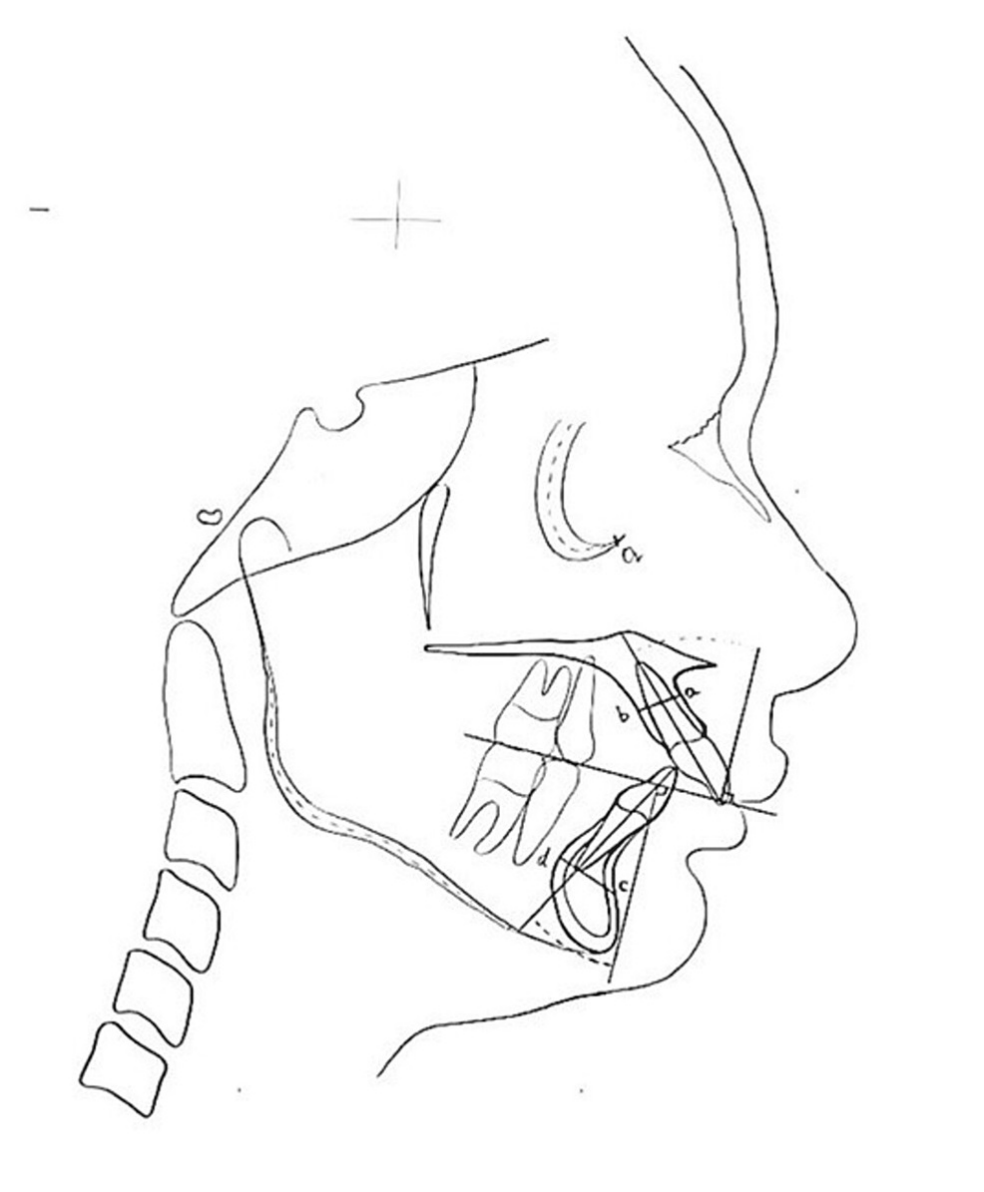
Measurements included (a) U1-lab, (b) U1-pal, (a+b) Mx-Alv, (c) L1-lab, (d) L1-ling, (c+d) Md-Alv, (e) U1-incl, (f) L1-incl, and (g) Wits Image Credits: Sumaiyya Haneen

Maxillary central incisor root position was measured in millimetres (mm) as the distance between the root's midpoint and the alveolar process's outer cortical surface on the labial (upper incisor - labial) and palatal (upper incisor - palatal) sides, perpendicular to the tooth's long axis. The total thickness of the maxillary alveolar process (Mx-Alv) was measured in millimetres (mm) by adding the distances between the upper incisor - labial aspect and the upper incisor - palatal aspect. Mandibular central incisor root position was measured in millimetres from the root apex to the outer cortical surface of the alveolar process on both labial (lower incisor-labial) and lingual (lower incisor-lingual) sides. The total thickness of the mandibular alveolar process (Md-Alv) was calculated by adding the distances between the lower incisor-labial and lower incisor - lingual. The inclinations of the maxillary (upper incisor - inclination) and mandibular (lower incisor - inclination) central incisors were measured using the angle produced between the long axis of the teeth and a line perpendicular to the occlusal plane (Figure 2). The AP-Jaw relationships were evaluated using the Wits assessment [8,9].

The study also employed the Sagittal Root Position (SRP) classification by Kan et al. [10], which divides root position within the bone into four divisions (classes I, II, III, and IV). All the angular and linear measurements were done manually by a single operator.

Statistical analysis

An adequate sample size was achieved by performing a power sample analysis. To achieve an 80% power level and a confidence interval of 90%, at least 10 participants/groups could be required for a total of 30 subjects.

All the statistical analyses were performed with the help of SPSS software version 21 (IBM Corp., Armonk, NY). Descriptive statistics were used to determine how individuals were distributed across the different categories. The Chi-square test determined dental arch form distribution based on sagittal root location. The ANOVA test was used to compare the groups' intergroup composition.

## Results

According to the sagittal root location and the A-P jaw relationship, descriptive statistics and a comparison of dental arch shapes were performed using the chi-square test and the ANOVA test, as shown in Tables 1-4. Table 1 shows the distribution of dental arch form according to SRP. Out of a total of 10 participants with class I SRP, six have a square arch form, two have a tapered form, and the other two have an oval arch form, whereas all the sample participants with class II SRP have a square dental arch form. Of the class III SRP participants, nine have a square arch form, one has a tapered form, and the other has an oval arch form. Sagittal root location and dental arch shape showed no statistically significant correlation (p = 0.311).

**Table 1:** Dental arch form distribution according to sagittal root position

	Oval form	Tapered form	Square form	Total	X^2^- value	p-value
Class I	6(60%)	2(20%)	2(20%)	10(100%)	4.773	0.311
Class II	9(100%)	0(0%)	0(0%)	9(100%)		
Class III	9(81.8%)	1(9.1%)	1(9.1%)	11(100%)		

**Table 2:** Dental arch form distribution according to classes of malocclusion

Arch shape	Class I	Class II	Class III
Oval	10 (100 %)	5 (50%)	9 (90%)
Tapered	0	3 (30%)	0
Square	0	2 (20%)	1 (10%)

**Table 3:** Descriptive and comparative analysis of the samples

Measurement	Class I		Class II		Class III		F-value	p-value
	Mean	Std. deviation	Mean	Std. deviation	Mean	Std. deviation		
U-labial	3.8500	0.88349	4.1500	1.15590	3.2500	0.97895	2.049	0.148
U-palatal	4.0000	0.94281	4.0500	0.49721	4.5000	0.97183	1.093	0.349
L-labial	2.9000	0.56765	3.7500	1.75198	3.1000	0.73786	1.505	0.024
L-lingual	3.4000	0.69921	3.3000	0.82327	3.5500	1.01242	0.217	0.035
Wits	1.1000	0.73786	7.4000	1.34990	−3.4000	1.26491	222.6	0.000^*^
U-angle	37.2000	7.17712	42.8000	8.18942	37.2000	6.42564	1.962	0.160
L-angle	34.1000	8.54335	31.5000	6.78642	16.6000	7.18331	15.676	0.000^*^

**Table 4:** Intragroup comparison for root positions

	Palatal/lingual		Labial		t-value	p-value
	Mean	SD	Mean	SD		
Maxillary (class I)	4.0000	0.94281	3.8500	0.88349	−0.537	0.026*
Mandibular (class I)	3.4000	0.69921	2.9000	0.56765	−3.000	0.015^*^
Maxillary (class II)	4.0500	0.49721	4.1500	1.15590	0.264	0.047*
Mandibular (class II)	3.3000	0.82327	3.7500	1.75198	0.850	0.035*
Maxillary (class III)	4.5000	0.97183	3.2500	0.97895	−3.822	0.004*
Mandibular (class III)	3.5500	1.01242	3.1000	0.73786	−1.221	0.023*

Table 2 shows the different grades of malocclusion affecting the distribution of dental arch forms. Of the 30 competitors, 24 had oval arches, three had tapering arches, and three had square arches. All the classes had an oval arch, but class II and class III participants only displayed a tapering arch and a square arch, respectively.

Table 3 displayed sample descriptive and comparative data across groups. Results showed that there was a highly statistically significant difference in AP jaw connection between the three classes (p=0.00). Classes II and III had higher mandibular incisor inclination, and there was a highly statistically significant difference between the three classes.

Table 4 showed an intragroup comparison of root positions. The difference between U-pal and U-lab and L-lab and L-lin was statistically significant in classes II and III.

## Discussion

Due to the fact that the main goal of orthodontic treatment is to rectify existing malocclusions, changes in arch dimensions may have an impact on the palate, which is regarded as a crucial anatomical element that influences skeletal patterns because of its form and position [11].

Lateral cephalograms are the most commonly used diagnostic tool. Because of its accessibility, low cost, low radiation exposure, and simplicity, the lateral cephalogram was used in this study over 3D imaging modalities [12].

The various archwires used in orthodontic treatment frequently change the proportions of the arches, which affects the stability of the maxillary and mandibular arches and the outcomes [13,14]. Oval arch forms were observed in all classes of malocclusion in the current investigation, but tapering arch forms were only observed in class II individuals, and square arch forms were only observed in class II and III subjects. In contrast, Kareem et al. examined the Kurdish population and reported that almost 90% of subjects had a tapered maxillary arch form, and 10% of them had an oval arch form [11]. The reason could be due to the ethnic background, as the present study was carried out in the south Indian population.

According to a recent Saudi study by Omar et al. [15], the thin, tapered, and narrow ovoid arch forms were the most common. The tapered shape of the arch was the most common maxillary arch form in both Malaysian and Indian populations, according to studies by Kareem et al. [16] and Othman et al. [17]. There were no squared maxillary arch forms among the Indian sample, and the bulk of them possessed tapering arch forms. While the majority of Malaysians had tapering arch forms, there were a very small number of people who had squared arch forms, and only a few who had oval maxillary arches. The ovoid maxillary arch type was the highest in a different study on the Malaysian population, followed by tapered and then square. Another person claimed that Indians frequently have narrow arches. The existence of many ethnic groups in other nations as well as regional ethnic diversity within India itself may be the cause of this enormous variance in outcomes [11,16].

In the present study, dental arch forms and sagittal root positions were evaluated in class 1, class 2, and class 3 patterns, as the intercanine width in the maxillary arch varies in different skeletal patterns. This is in accordance with a study by Sharoudi and Etezadi, who concluded that a strong positive correlation existed between SNA angle and upper intercanine width, which meant that patients with a higher SNA angle had a wider dental arch in the canine region [18].

In this current study, the variation was not statistically significant in the distribution of dental arch form based on sagittal root location (p > 0.05). Class IV SRP was not found in any skeletal pattern. According to a study by Somvasoontra et al., this was true [6]. They noted that there was no correlation between tooth arch shape and SRP. This finding implies that the SRP categorization could not be predicted using just the dental arch morphology.

The present study showed the intergroup descriptive and comparative data of the samples. There was a highly statistically significant difference between the three classes in terms of the AP-Jaw relationship (p < 0.05). Mandibular incisor inclination was found to be higher in classes II and III, and the difference between the three classes was highly statistically significant. This was in accordance with a study done by Andrews et al. [9], who found similar results. They discovered that class II patients' mandibular central incisors displayed labial tilting as a result of positioning adjustment for pre-existing antero-posterior skeletal abnormalities.

In the current investigation, the difference between U-pal and U-lab and L-lab and L-lin in the intragroup comparison of root locations for the mandibular class II and maxillary class III was statistically significant. This was in line with research by Tian et al. [19], Gracco et al. [20], and Andrews et al. [9].

These results imply a correlation between class I, II, and III malocclusions, sagittal root locations, and alveolar bone thickness. However, there was no correlation between the dental arch shape and sagittal root position. Therefore, it is important to recognise the therapeutic value of these data and make use of them when developing an orthodontic treatment plan.

With the understanding that altering these naturally existing ideal root locations might make up for incorrect AP jaw position discrepancies or help with facial profile aesthetics, these findings can be used as a reference point for diagnosis and treatment planning. However, these variations might endanger the roots' and/or supporting tissues' structural integrity. Planning for rapid implant implantation at the location of the maxillary central incisor may be aided by bone volume prediction that takes these considerations into account. This would lead to long-term success and improved aesthetic results.

As a result of the study's limited sample size, it may have some limitations. Therefore, additional research should be done to assess the relationship between alveolar bone thickness, sagittal root positions, and arch forms in classes I, II, and III malocclusions in a more varied population with age considerations as well for an accurate assessment and detailed evaluation.

## Conclusions

The result of the current study led to the conclusion that the dental arch form distribution according to classes of malocclusion showed that an oval arch form was seen in all the classes, whereas a tapered arch form was seen only in class II subjects and a square arch form was seen in class II and III subjects. Classes II and III were found to have higher mandibular incisor inclinations. In the mandibular class II and maxillary class III root locations, there was a substantial difference between U-pal and U-lab, L-lab, and L-lin. No association was found between the dental arch form and the sagittal root position. Hence, advanced research is essential to address factors specific to sagittal root position and dental arch form and to assess various parameters on a larger scale.
